# Differentiation capacities of skeletal muscle satellite cells in Lantang and Landrace piglets

**DOI:** 10.18632/oncotarget.17860

**Published:** 2017-05-15

**Authors:** Chun-Qi Gao, Yin-Long Xu, Cheng-Long Jin, Xiao-Chao Hu, Hai-Chang Li, Guang-Xu Xing, Hui-Chao Yan, Xiu-Qi Wang

**Affiliations:** ^1^ College of Animal Science, South China Agricultural University/Guangdong Provincial Key Laboratory of Animal Nutrition and Regulation/ National Engineering Research Center for Breeding Swine Industry, Guangdong, China; ^2^ Guangzhou United Bio-Technology Feed Co., Ltd, Guangzhou, China; ^3^ Davis Heart & Lung Research Institute, Wexner Medical Center at the Ohio State University, Columbus, OH, USA; ^4^ Key Laboratory of Animal Immunology of the Ministry of Agriculture, Henan Academy of Agricultural Sciences, Zhengzhou, China

**Keywords:** satellite cells, differentiation capacity, mTOR signaling pathway

## Abstract

We isolated and cultured satellite cells (SCs) from the longissimus dorsi muscles of 1-day-old male Landrace and Lantang piglets to compare the SC differentiation capacity in the two breeds. Lantang piglets yielded more (*P* < 0.05) SCs per gram of muscle than Landrace piglets (5.2 ± 0.9×10^4^ vs. 2.4 ± 0.2×10^4^). Transcription of the differentiation markers myogenin and myosin heavy chain I (MyHC I) in the longissimus dorsi muscle was higher in Lantang than Landrace piglets (*P* < 0.05). Protein levels of myogenin (*P* < 0.05), MyHC I (*P* < 0.05), and myogenic regulatory factor 4 (*P* = 0.07) were higher in Lantang than Landrace piglet SCs after 72 h of differentiation. Creatine kinase activity was higher in Lantang than Landrace piglet SCs after 24, 48, and 72 h of differentiation (*P* < 0.05), and there was a greater fusion index in Landrace piglet SCs after 72 h of differentiation. In addition, phosphorylation of Akt, mTOR, S6K1, S6, and 4EBP1 was lower in Lantang than Landrace piglet SCs (*P* < 0.05). Thus differentiation was more extensive in Lantang than Landrace piglet SCs, but expression of the mTOR signaling pathway was lower in Lantang piglet SCs, suggesting mTOR signaling may inhibit myogenic differentiation. These findings reveal that mTOR signaling is a factor in myogenesis and imply that mTOR could potentially serve as an activator of myoblast differentiation and muscle regeneration.

## INTRODUCTION

As muscle stem cells, satellite cells (SCs) promote myofiber growth and damage repair through proliferation and differentiation [1–2]. Approximately 60% to 70% of adult mammalian muscle DNA accumulates after birth, and SC differentiation is a factor [3]. During the process of skeletal myogenesis, SCs exit the cell cycle and fuse to form multinucleated myotubes through differentiation, and finally participate in the process of skeletal muscle hypertrophy [4]. After the muscle differentiation process, adult skeletal muscle responds to physical injuries or muscular disorders by activation and proliferation of SCs [5].

In the myogenic differentiation process numerous signaling pathways promote the formation of skeletal muscle [6]. The basic helix-loop-helix proteins and the mammalian target of rapamycin (mTOR) signaling pathway are promotors of skeletal myogenesis and the multiple stages of myogenic differentiation through distinct mechanisms [7–8]. Le Grand and Rudnicki [9] indicated that the expression of myogenin, myogenic regulatory factors 4 (MRF4), myosin heavy chain I (MyHC I), and myogenic differentiation antigen are activated and controlled during differentiation. Apart from basic helix-loop-helix proteins, mTOR acts as a nutrient signal transducer sensing the extracellular growth factors and amino acids to stimulate cell activity and myogenic differentiation [10–11]. The biochemical complex mTORC1 is sensitive to rapamycin, and it initiates translation via the 70-kDa ribosomal protein S6 kinase 1 (S6K1) and eukaryotic translation initiation factor 4E-binding protein 1 (4E-BP1) [12]. mTOR complexes are required for phospholipase D-mediated myogenic differentiation [13]. The inhibition of mTOR kinase prevents S6K1 activity, and then suppresses C2C12 myoblast differentiation [7]. Some studies suggest that mTOR suppresses myogenic differentiation [12]. Neither knockdown nor overexpression of S6K1 or 4E-BP1 has any effect on myogenic differentiation [6, 14]. Therefore, the function of the mTOR signaling pathway in myogenic differentiation is not clear, even though the pathway is suggested to control skeletal myogenesis at multiple stages.

Different breeds of swine are known to have predetermined propensities in certain areas, such as carcass composition and meat quality, which are associated with SC proliferation and differentiation [15–18]. Lantang pigs are native to Southern China and characterized by better meat quality and disease resistance compared with Landrace pigs. Our studies have shown that Lantang piglet SCs are more proliferative than Landrace piglet SCs, and the mTOR signaling pathway stimulates the SC proliferation [19]. In addition, the myofibers in Lantang piglets have a higher density but smaller cross-sectional area compared with Landrace piglets. Therefore, the different muscle characteristics might be related to the different features of skeletal muscle SCs, such as differentiation. To examine the relation between SC differentiation and muscle fiber traits, 1-day-old Lantang piglets and Landrace piglets were used to compare the SC differentiation capacity, as well as the mTOR pathway to investigate the molecular mechanisms.

## RESULTS

### Body weight of piglets and number of SCs harvested from longissimus dorsi muscle

**Figure 1:**
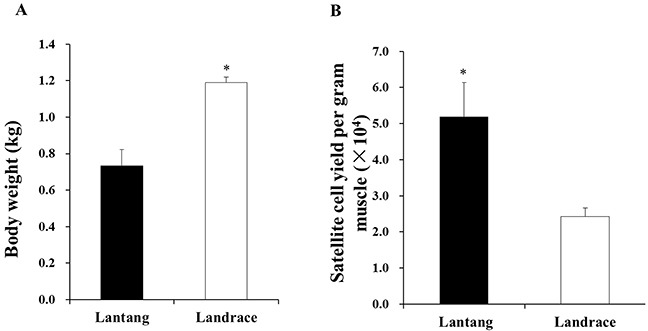
The body weight **(A)** and satellite cells (SCs) yield (number/gram of longissimus dorsi muscle) **(B)** from 1-day-old Lantang and Landrace piglets, respectively. Asterisk (*) indicates a significant difference (P < 0.05).

The 1-day-old Lantang and Landrace piglets were weighed before being sacrificed (Figure 1A). The Landrace piglets had higher (*P* < 0.05) body weights than the Lantang piglets. The Lantang piglet muscle samples yielded more (*P* < 0.05) SCs per gram of muscle than the Landrace piglet muscle samples (5.2 ± 0.9×10^4^ cells versus 2.4 ± 0.2×10^4^ cells/gram of muscle) (Figure 1B).

### mRNA content of myogenin and MyHC I in longissimus dorsi muscle

**Figure 2:**
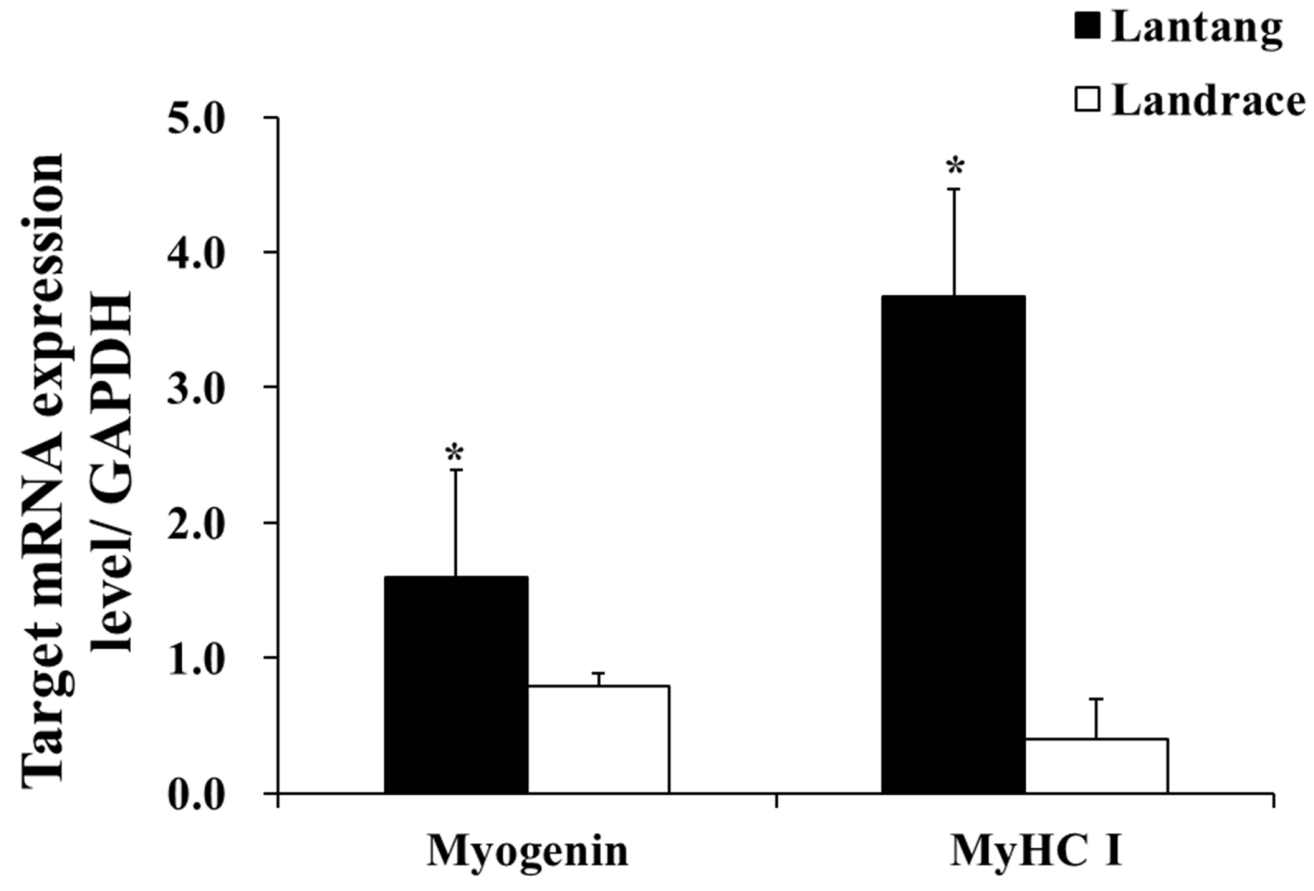
mRNA content of myogenin and myosin heavy chain I (MyHC I) in longissimus dorsi muscle of 1-day-old Lantang and Landrace piglets The values were normalized by glyceraldehyde-phosphate dehydrogenase (GAPDH) content. Values are mean ± SEM. Asterisk (*) indicates a significant difference (P < 0.05).

To directly compare the differentiation capacities of muscle SCs in Lantang and Landrace piglets, the relative mRNA content of myogenin and MyHC I in Lantang and Landrace piglet longissimus dorsi muscle was determined (Figure 2). The Lantang piglet SCs had greater differentiation capacity than Landrace piglet SCs, as indicated by higher mRNA content of the early myogenic marker myogenin and the later myogenic marker MyHC I (*P* < 0.05) (Figure 2).

### Creatine kinase activity and fusion index of SCs during differentiation

**Figure 3:**
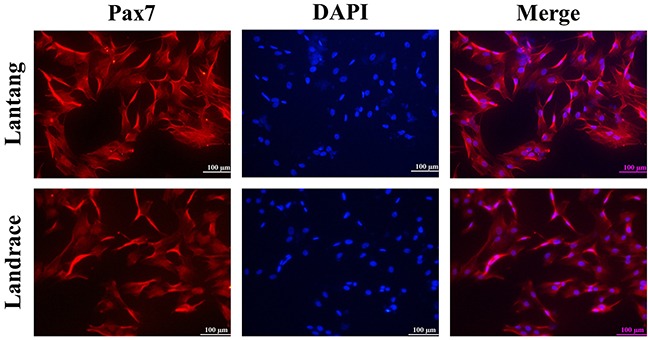
Identification by immunofluorescence staining of satellite cells (SCs) The images represent Lantang piglet and Landrace piglet SCs immunolabelled with anti-human paired box protein (Pax7) polyclonal antibody, respectively. Pax7 immunostaining is depicted in red. Nuclei are stained with DAPI (blue). Scale bar: 100 μm.

**Figure 4:**
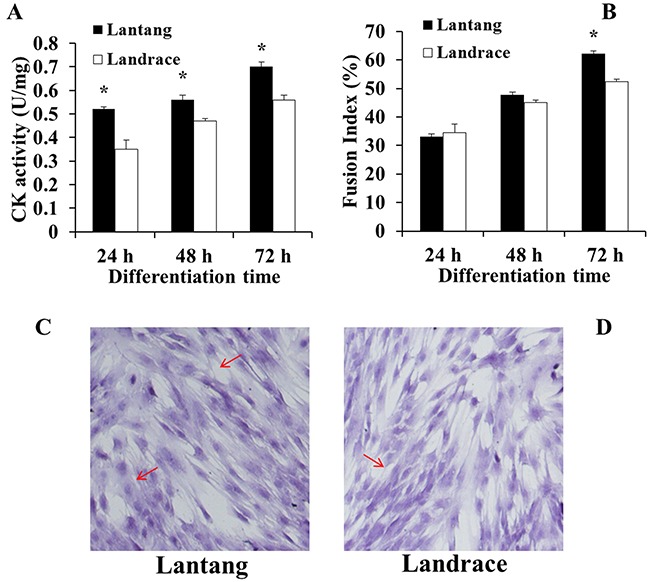
Creatine kinase (CK) activity **(A)** and fusion index **(B)** of satellite cells (SCs) from Lantang and Landrace piglets were measured every 24 hours during differentiation in Dulbecco's modified Eagle's medium (DM). CK activity **(A)** was calculated by normalizing to total protein content. Fusion index **(B)** was examined by hematoxylin eosin staining (HE) method (**C**, Lantang; **D**, Landrace). The original magnification was 100×. Data represent means ± SEM. Asterisk (*) indicates a significant difference (*P* < 0.05).

After SCs were isolated, purified, and identified (Figure 3), the activity of creatine kinase (CK) was measured to evaluate the differentiation capacity of SCs. Compared with Landrace piglet SCs (Figure 4A), the CK activity in Lantang piglet SCs was higher (*P* < 0.05) at 24, 48, and 72 hours during differentiation. Myotube formation resulting from differentiation was assessed every 24 hours after initiation of differentiation by measuring the fusion index. Data showed that SCs from Lantang piglets had a higher (*P* < 0.05) fusion index than that in Landrace piglets at 72 hours (Figure 4B).

### MRF4, myogenin, and MyHC protein content at 72 hours during differentiation

**Figure 5:**
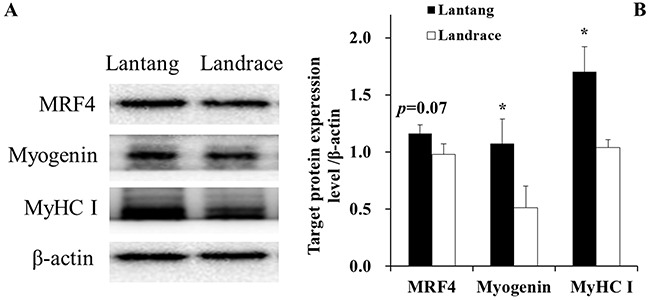
Protein levels of myogenic regulatory factor 4 (MRF4), myogenin, and myosin heavy chain I (MyHC I) in Lantang and Landrace satellite cells (SCs) Western blotting analyzed for MRF4, myogenin, MyHC I in satellite cells at 72 hours during differentiation **(A)**. The values were normalized by β-actin expression **(B)**. Values are mean ± SEM. Asterisk (*) indicates a significant difference (*P* < 0.05).

To further study the SC differentiation, the protein expression of muscle differentiation markers, including MRF4, myogenin and MyHC, were also examined at 72 hours during differentiation (Figure 5). The results showed that SCs from Lantang piglets have a higher (*P* = 0.07) expression of MRF4, myogenin, and MyHC proteins than SCs from Landrace piglets (Figure 5).

### mTOR signaling expression at 72 hours during differentiation

**Figure 6:**
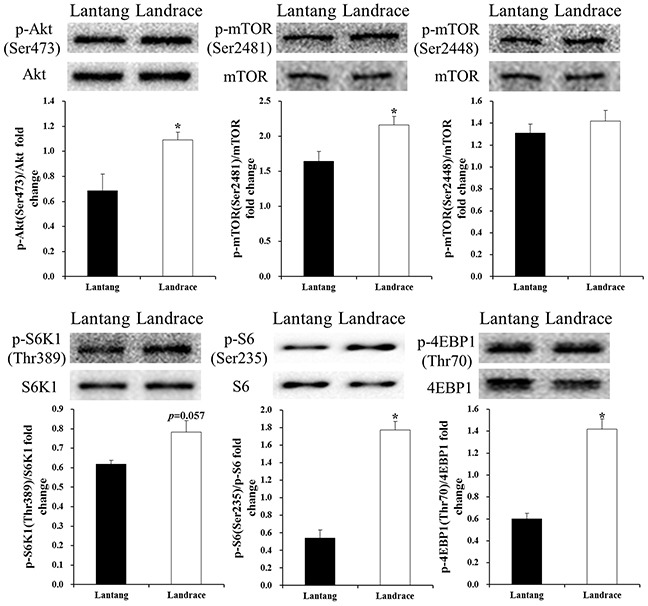
Protein levels of mammalian target of rapamycin (mTOR) pathway regulators in Lantang and Landrace piglet satellite cells (SCs) Western blot analysis of satellite cells for protein kinase B (Akt), p-Akt (Ser437), mTOR, p-mTOR (Ser2448, Ser2481), p70 ribosomal S6 protein kinase 1 (S6K1), p-S6K1(Thr389), ribosomal protein S6 (S6), p-S6 (Ser235/236), eukaryotic translation initiation factor 4E-binding protein 1 (4EBP1), and p-4EBP1(Thr70) at 72 hours during differentiation. The results show phosphorylation levels relative to total levels. Values are mean ± SEM. Asterisk (*) indicates a significant difference (*P* < 0.05).

To examine the role of the mTOR pathway during myoblast differentiation, the expression of total protein levels and phosphorylation ratios of Akt, mTOR, S6K1, and S6, as well as 4EBP1, were determined (Figure 6). Results revealed that the levels of p-Akt (Ser473), p-mTOR (Ser2481), p-S6 (Ser 235/236), and p-4EBP1 (Thr70) in Lantang piglet SCs were lower (*P* < 0.05) than levels in Landrace piglet SCs, and the level of p-S6K1 (Thr389) was lower (*P* = 0.057) than the level in Landrace piglet SCs. However, the protein levels of p-mTOR (Ser2448) in Lantang piglet SCs showed no difference compared with Landrace piglet SCs.

## DISCUSSION

Muscle damage repair is influenced by myofiber growth, and the muscle fiber characteristics (fiber diameter and density) are inseparable from SC functions [18, 20]. Previous studies proved that SCs represent 2.5% to 6% of nuclei in one muscle fiber, indicating the importance of SCs for muscle fiber repair and hypertrophy [2, 4, 21]. A decrease in SC number can lead to muscle cachexia and atrophy [22]. Our present study showed that the yield of SCs from 1-day-old Lantang piglet longissimus dorsi muscle was higher than from Landrace piglets. Our previous study found that the density of muscle fiber in Lantang piglet longissimus dorsi muscle was higher than in Landrace piglets [19]. Therefore, we hypothesized that the higher number of SCs contributes to the higher density of muscle fibers, and the Lantang piglet SCs have a superior repair ability to that of Landrace piglet SCs when myofibers are damaged.

The processes by which SCs fuse into myotubes consists of a series actions, including SC proliferation and differentiation [23]. The basic helix-loop-helix proteins, including the early myogenic marker myogenin, MRF4, and the later myogenic marker MyHC, block cell cycle progression by causing G1 arrest, myogenic differentiation, and myotube maturation [9, 24]. Cell differentiation is promoted by the basic helix-loop-helix proteins and myokines such as upregulated IL-15 [25–26]. We found that myogenin and MyHC I mRNA content in Lantang piglet longissimus dorsi muscle were higher than in Landrace piglets. The expression of myogenin, MRF4, and MyHC I protein levels in Lantang piglet SCs were higher than in Landrace piglet SCs. These results indicate that Lantang piglet SCs might have greater differentiation capacity than Landrace piglet SCs.

To further validate the hypothesis, we examined the CK activity and the fusion index in Lantang piglet and Landrace piglet longissimus dorsi muscle SCs. CK is an index for measuring skeletal muscle SC differentiation capacity [27–28]. In slow muscle, the interaction between mitochondria CK and muscle cytoplasm CK could form a phosphocreatine-creatine cycle that can transfer ADP and ATP between mitochondria and sites of energy expenditure. Our results showed that CK activity in Lantang piglet SCs is greater than in Landrace piglet SCs during differentiation. The results indicated that Lantang piglet SCs possess a greater differentiation capacity compared with Landrace piglet SCs.

SCs that fuse to existing fibers or form myotubes appear during muscle growth and repair [29]. Myotube formation *in vitro* represented the muscle fibers process [30]. The fusion index is an indicator of myotube formation, the quantification of the fusion index is related to SC myotube formation and differentiation capacity [31]. We found that SCs from Lantang piglets have a higher fusion index than SCs from Landrace piglets during differentiation at 72 hours, indicating that the differentiation capacity of Lantang piglet SCs is greater than that of Landrace piglet SCs. In addition, our previous study found that the myofibers in Lantang piglets have a higher density compared with myofibers in Landrace piglets [19]. Considering the different fiber density between the two breeds, we hypothesized that the difference was caused by the different fusion or differentiation capacity of SCs. Taken together, the results of these *in vivo* and *in vitro* experiments suggest that the Lantang piglet SCs have greater differentiation capacity than Landrace piglet SCs.

Previous studies suggest that the mTOR signaling pathway is crucial in cell growth, differentiation, and regeneration, and has long been recognized as a factor in skeletal muscle growth [32–34]. However, the exact function of the mTOR pathway in muscle cell differentiation and regeneration is ambiguous. Yoon and Chen [6] found that mTOR activation can promote phosphorylation of insulin receptor substrate-1 and suppress cell differentiation of mouse C2C12. Similarly, Ge et al [12] indicated that mTOR inhibits C2C12 myogenic differentiation by suppressing PI3K/Akt signaling. Moreover, Jayaraman and Marks [35] demonstrated that rapamycin, the mTOR kinase inhibitor, blocks proliferation and induces differentiation of BC1H1 mouse cells. Consistent with these previous studies, we found that the protein expression of p-Akt (Ser473), p-mTOR (Ser2481), p-S6K1 (Thr389), p-S6 (Ser 235/236), and p-4EBP1 (Thr70) in Lantang piglet SCs were lower than in Landrace piglet SCs, whereas the Lantang piglet SCs exhibited more myogenic differentiation compared with Landrace piglet SCs. Therefore, our findings and the above observations suggest that the mTOR signaling pathway has opposing functions in myogenic differentiation.

However, Jaafar et al [13] reported that the mTOR complexes were required for phospholipase D activated myogenic differentiation [13]. The inhibition of mTOR kinase suppresses C2C12 myoblast differentiation [7]. Furthermore, Gardner et al [36] found that deletion of Akt disrupts the normal process of cell differentiation, and similar results were found in the study of Briata et al [5]. The different responses of the mTOR signaling pathway in muscle differentiation in different experiments can be explained by the differences in the stages of myogenesis. During skeletal myogenesis, the mTOR signaling pathway is not needed at the initial stage of differentiation, whereas it is required for late-stage fusion leading to the myotube and myofiber maturation [37–38]. Additionally, the protein expression pattern of the mTOR signaling pathway might differ in various animal species. However, the mechanism through which the mTOR signaling pathway exerts its effects on muscle differentiation is yet to be investigated.

Lantang piglet SCs have a greater differentiation capacity than Landrace piglet SCs, leading to a different expression pattern of proteins on the mTOR signaling pathway. Thus, mTOR kinase might be utilized to stimulate muscle differentiation and regeneration.

## MATERIALS AND METHODS

### Porcine muscle collection, SCs isolation, and culture

This study was conducted with the approval and in accordance with the directives of the Institutional Animal Care and Use Committee of South China Agricultural University (Guangzhou, China).

Six 1-day-old male Lantang piglets (BW: 0.73±0.09 kg) and six 1-day-old Landrace male piglets (BW: 1.19±0.03 kg) were used in this study. The piglets were euthanized with sodium pentobarbital before sampling. Longissimus dorsi muscle was used to isolate SCs. Additional muscle samples were taken and frozen in liquid nitrogen immediately, and then stored at −80°C until analysis.

The method used to isolate SCs was previously described [17, 39, 40], and immunocytochemistry was used to identify SC purity by paired box protein 7 (Pax7). Immunocytochemistry analysis showed the positive cells for the Pax7 polyclonal antibody (Wuhan Huamei Biotech Co. Ltd, Wuhan, China) accounted for 90% of total cells (Figure 3). Cells were cultured in a growth medium consisting of Dulbecco's modified Eagle's medium (DMEM) supplemented with 10% (v/v) fetal bovine serum (FBS) and antibiotics (100 IU/mL penicillin, 100 μg/mL streptomycin) in 5% carbon dioxide (CO_2_) at 37°C. Cell culture flasks and plates were obtained from Corning (Corning, NY, USA).

### SC yield

Cells were counted by use of a hemocytometer according to the method of Zhu et al [41]. Cell yield was calculated by comparing the total number of cells with the wet weight of the muscle samples.

### Cell myogenic differentiation

For myogenic differentiation, SCs were cultured in six-well plates at greater densities (2.6 × 10^5^ cells/cm^2^) to adjust for the different proliferation. Once attached to the plate (85% to 90% confluence), cultures were induced to differentiate by use of differentiation medium (DM) containing 2% horse serum. DM was changed every 24 hours for 72 hours. After the addition of the DM, three randomly selected plates were rinsed three times with cold sterile 1× phosphate buffered saline (PBS) and fixed at 4°C with 4% paraformaldehyde every 24 hours over a 3-day period. We analyzed cells morphologically by hematoxylin and eosin (H&E) staining. Fifteen randomly selected fields were enumerated for total and fused nuclei. Fused nuclei were defined as any two or more nuclei within a continuous membrane-defined myotube. The percentage of myonuclei contained in myotubes, as indicated by the fusion index, was calculated.

### Creatine kinase activity assay

Every 24 hours to 72 hours of differentiation, cell culture plates were removed from the incubator, rinsed three times with cold sterile 1 × PBS and immediately lysed in ice-cold lysis buffer. Lysates were centrifuged at 13,000 × g for 15 minutes at 4°C, the supernatants were collected, and the protein content of the samples were measured by use of Bicinchoninic Acid Protein Assay Kit (BCA, Thermo Fisher Scientific, San Jose, CA, USA). CK activity was measured by Autobiochemical Analyzer (SYSMEX, Kobe, Japan) according to the manufacturer's instructions. Specific CK activity was calculated by normalizing to total protein content, and differentiation was determined by measuring the muscle-specific CK activity.

### RNA isolation and real-time polymerase chain reaction

RNA was isolated from Lantang and Landrace piglet longissimus dorsi muscle with Trizol reagent (Invitrogen, Carlsbad, CA). Quality of RNA was assessed by agarose gel electrophoresis and OD_260_/OD_280_ ratios between 1.8 and 2.0. Reverse transcription was performed by use of Moloney Murine Leukemia Virus Reverse Transcriptase (Invitrogen, Carlsbad, CA). Synthesis of the cDNA first strand was performed with random primers (N10) and Superscript II reverse transcriptase (Invitrogen, Carlsbad, CA).

The mRNA content (n = 6) was determined by real-time polymerase chain reaction (PCR) using a One-Step SYBR Green PCR Mix (Takara, Dalian, China). Primers were designed specifically for each gene by Primer 5.0 software (Table 1). Amplification and melting curve analysis were performed on a Stratagene Mx3005P real-time PCR system (Stratagene, La Jolla, CA). Sizes of products were verified by electrophoresis on ethidium bromide-stained 1.0% agarose gels in Tris acetate-EDTA buffer. Relative mRNA expression level was calculated by application of the 2^−ΔΔCt^ method with glyceraldehyde-phosphate dehydrogenase (GAPDH) as the housekeeping gene.

**Table 1:** Primers used for quantitative real-time PCR

Genes (accession no)	Primers	Sequences (5′–3′)	Size (bp)
Myogenin	sense	GTCCCCAGCCCCTTATCTT	138
	antisense	GATTATCACGCTACGACGGA	
MyHC I	sense	GAGAAGGGCAAAGGCAAGG	118
	antisense	ACGAAGTGGGGATGTGTGG	
GAPDH	sense	GGTCGGAGTGAACGGATTTG	170
	antisense	CCTTGACTGTGCCGTGGAAT	

### Protein extraction and Western blot analysis

SCs were washed for three times with cold PBS, and then lysed on ice with lysis buffer (BioTeke, Beijing, China). Subsequently, the samples were centrifuged at 12,000× g for 5 minutes at 4°C to remove insoluble debris. The supernatants were used for the experiments, and the protein concentrations were determined by use of the BCA method (Thermo Fisher Scientific, San Jose, USA). Samples mixed with an electrophoresis sample buffer were boiled for 10 minutes at 100°C.

An equal amount of protein was electrophoresed on a 10% SDS polyacrylamide gel in a tris-glycine running buffer. After the electrophoresis was completed, all the proteins were transferred to polyvinylidene difluoride membranes (Bio-Rad Laboratories, Hercules, CA, USA) at 100 V for 75 minutes by use of a transfer buffer. The membranes were blocked with 5% BSA in tris-buffer saline for 2.5 hours at room temperature, and then incubated overnight at 4°C with primary antibodies. After incubation with the first antibody and extensive washing, membranes were incubated with the appropriate horseradish peroxidase-labeled second antibody for 1.5 hours at 26°C. Proteins reacting with the first antibody were visualized with the ECL-Plus Western blotting reagent in a FluorChem M system (Cell Biosciences, San Leandro, CA, USA). Band density was analyzed by Image J (http://rsb.info.nih.gov/ij/).

The primary antibodies, anti-Akt (9272S), anti-phospho-Akt (9271S, Ser437), anti-mTOR (2972S), anti-phospho-mTOR (5536S, Ser2448; 2974S, Ser2481), anti-S6K1 (9202S), anti-phospho-S6K1 (9205S, Thr389), anti-S6 (2708S), anti-phospho-S6 (9234S, Ser235/236), anti-4EBP1 (9644S), anti-phospho-4EBP1 (2855S, Thr70), and anti-β-actin, were purchased from Cell Signaling Technology (Beverly, MA, USA). Anti-MRF4 (sc-301), anti-Myogenin (sc-31943), anti-MyHC (sc-20641), and anti-Myostatin (sc-28910) were purchased from Santa Cruz Biotechnology (Santa Cruz, CA, USA). Second antibody anti-rabbit IgG was purchased from Beijing Biosynthesis Biotechnology Co. Ltd (Beijing, China).

### Statistical analysis

Data are expressed as the means ± SE. Student's *t*-test was conducted to determine differences between two groups by use of SAS (Version 9.2; SAS Institute Inc., Cary, NC). The asterisk (*) indicates a significant difference (P < 0.05). The results are representative of three separate experiments.
